# Association between umbilicus–symphysis pubis distance and operative time in transabdominal preperitoneal (TAPP) inguinal hernia repair

**DOI:** 10.1007/s10029-026-03687-7

**Published:** 2026-04-29

**Authors:** Emre Gülçek, Yunushan Furkan Aydoğdu, Safa Özaydın, Çağrı Büyükkasap

**Affiliations:** 1 Department of General Surgery, Çanakkale Onsekiz Mart University Faculty of Medicine, Çanakkale, Türkiye; 2 Department of General Surgery, Ankara Training and Research Hospital, Ankara, Türkiye; 3 Department of General Surgery, Kırıkkale High Specialization Hospital, Kırıkkale, Türkiye; 4 Department of General Surgery, Gazi University Faculty of Medicine, Ankara, Türkiye

**Keywords:** TAPP, Laparoscopic inguinal hernia repair, Umbilicus–pubic symphysis distance, Anatomical factors, Patient selection

## Abstract

**Background:**

The transabdominal preperitoneal (TAPP) approach is a widely used minimally invasive technique for laparoscopic inguinal hernia repair. However, operative time and technical difficulty may vary depending on patient-related anatomical factors. This study evaluated the effect of the umbilicus–symphysis pubis (USP) distance on operative time and investigated whether this superficial anatomical measurement could guide patient selection.

**Methods:**

In this retrospective study, data from 767 patients who underwent TAPP repair at Gazi University Hospital between January 2015 and June 2022 were reviewed. After applying the exclusion criteria, 341 male patients were included. Of these, 272 had unilateral and 69 had bilateral inguinal hernia. The distance between the umbilicus and symphysis pubis was measured preoperatively in all patients. The relationship between USP distance and operative time was analyzed using the Python-based Maximally Selected Rank Statistics (MaxStat) method, and optimal cut-off values were determined.

**Results:**

The age range of the study group was 18.00–77.00 years, with a median age of 44.00 years. The USP distance ranged from 9.70 to 23.50 cm in unilateral cases and from 11.10 to 22.30 cm in bilateral cases. MaxStat analysis identified optimal cut-off values of 15 cm for unilateral and 16 cm for bilateral hernia cases. In unilateral cases, operative time was significantly longer in patients with a USP distance ≤ 15 cm (*p* < 0.001). Similarly, in bilateral cases, operative times were significantly longer when the USP distance was ≤ 16 cm (*p* < 0.001).

**Conclusion:**

USP distance may serve as a simple and easily measurable anatomical parameter associated with surgical duration, particularly in unilateral TAPP repairs. As patients with a shorter USP distance were associated with longer operative times, this association warrants further prospective validation before USP distance can be considered a routine parameter in preoperative planning.

## Introduction

The Transabdominal Preperitoneal (TAPP) approach in laparoscopic inguinal hernia repair has become an increasingly preferred technique with the development of minimally invasive surgery. However, operative time and technical difficulty in TAPP repair may vary depending on multiple factors, including patient-related anatomical characteristics. During this process, numerous variables, such as case selection, operation duration, and complication rates, are of great importance in terms of both patient safety and the sustainability of surgical success [1–3]. The literature has shown that, in addition to the surgeon’s experience, certain anatomical factors specific to the patient may also influence the technical difficulty of the operation. These factors are not limited to inguinal hernia surgery but also play an important role in other laparoscopic procedures involving the pelvic region. In such procedures, anatomical limitations can directly affect the surgeon’s field of view, instrumentation movement capability, and ergonomics [4–6].

In the TAPP approach, the surgeon’s working area is defined within the limited anatomical structure of the lower abdomen. In this context, it is anticipated that superficial anatomical parameters, such as the distance between the umbilicus and the symphysis pubis (USP), may influence operative conditions and duration. A short USP distance may cause ergonomic difficulties by restricting the surgeon’s range of motion, particularly during laparoscopic trocar placement and instrument manipulation. This situation may lead to prolonged operative times, increased technical errors and, indirectly, an elevated risk of complications. However, it should be emphasized that surgical ergonomics is influenced by multiple factors, including trocar positioning, working angles, and overall anatomical working space. Furthermore, such anatomical constraints may increase operative complexity and contribute to prolonged operative times, regardless of surgeon experience level [7–9].

In this study, the effect of the USP distance on the duration of TAPP procedures was examined, and it was investigated whether this anatomical measurement could serve as a preoperative parameter to support surgical planning.

## Methods

This retrospective study was conducted by reviewing data from TAPP inguinal hernia repairs performed at Gazi University Hospital between January 2015 and June 2022. A total of 767 patients, aged 18–99 years, operated on by four senior surgeons with established surgical practice, were included in the study.

**Fig. 1:**
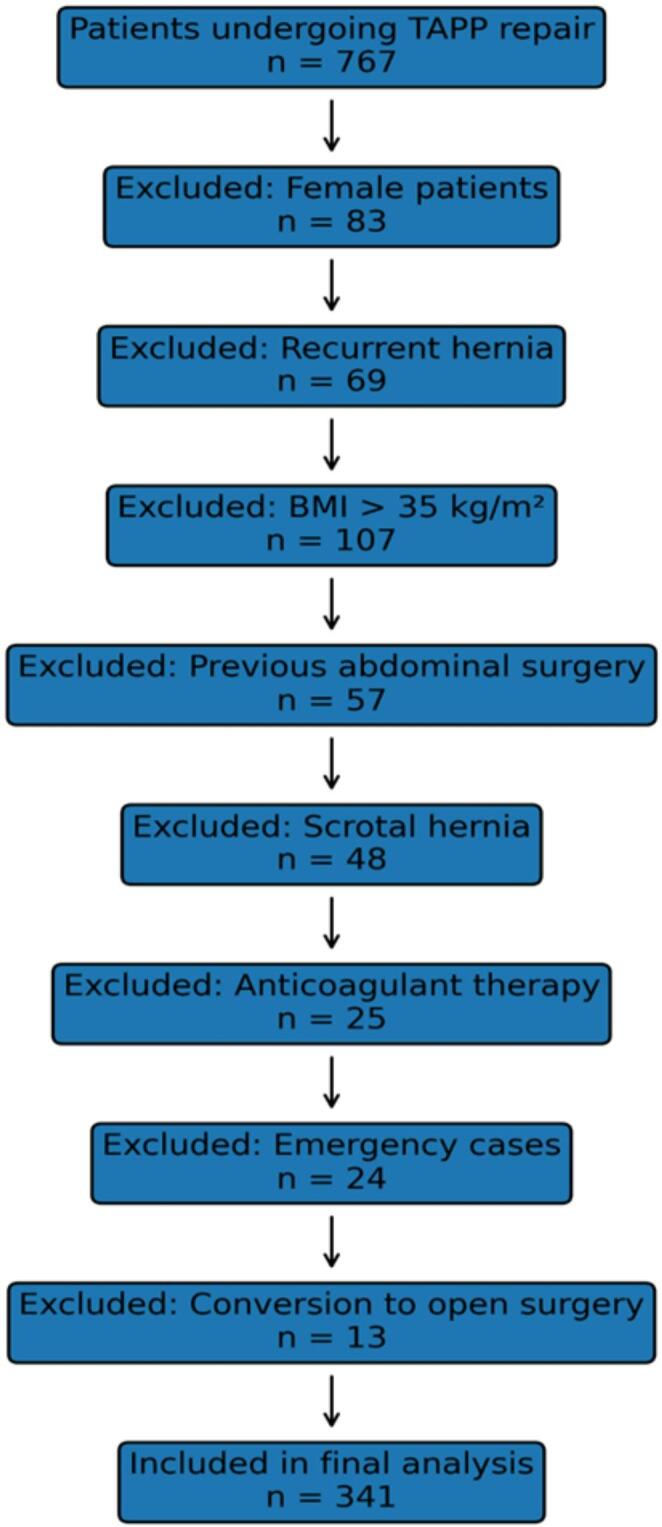
Patient selection flow diagram

The following patient groups were excluded from the study according to the defined exclusion criteria: female patients (*n* = 83), those diagnosed with recurrent inguinal hernia (*n* = 69), those with a body mass index (BMI) > 35 kg/m² (*n* = 107), those with a history of abdominal surgery (*n* = 57), patients with scrotal hernia (*n* = 48), those undergoing anticoagulant therapy (*n* = 25), patients requiring emergency surgery (*n* = 24), and cases converted to open surgery due to intraoperative complications (*n* = 13), as well as patients diagnosed with femoral hernia (Fig. 1).

Following the application of these criteria, a total of 341 patients were included in the study. Of these patients, 272 underwent surgery for unilateral inguinal hernia and 69 for bilateral inguinal hernia.

For each patient, the distance between the umbilicus and the symphysis pubis (USP) was recorded prior to surgery according to a standard measurement protocol. The measurement was performed while the patient was in the supine position, by measuring the shortest linear distance between the center of the umbilicus and the palpated upper border of the symphysis pubis in centimeters, using a non-stretchable tape measure over the skin. These measurements were analyzed by grouping the cases according to the presence of unilateral or bilateral hernias. The USP distance was evaluated as a potential anatomical marker for predicting operative duration and potential intraoperative challenges of the operation that the surgeon might encounter during surgery.

### Surgical technique

Pneumoperitoneum was created using the Veress needle technique applied to the infraumbilical region. A 10 mm port was placed for entry. The abdomen was insufflated at 14 mmHg pressure, and the patient was placed in the 30° Trendelenburg (head-low) position to allow better visualization of the pelvic region.

Subsequently, both groin regions were carefully evaluated to investigate the presence of hernias. To enable more effective execution of the procedure, two additional 5 mm ports were placed along the right and left midclavicular lines at the level of the umbilicus; in bilateral cases, ports were positioned symmetrically, whereas in unilateral cases, the contralateral port was placed slightly lower than the ipsilateral side to improve ergonomic access. Subsequently, a peritoneal incision was made extending from the medial umbilical plica to the lateral cephalic myopectineal orifice. Dissection was performed in the Retzius and Bogros areas, and important anatomical structures such as the epigastric vessels, Cooper’s ligament, corona mortis, and external iliac vessels were identified and exposed.

Dissection of the hernia sac was performed carefully, taking care to preserve the spermatic fascia. Subsequently, a polypropylene patch (Prolene^®^ Mesh, Ethicon, USA) measuring 8 × 12 × 15 cm was placed over the relevant anatomical structures and secured with absorbable staples (AbsorbaTack™, Medtronic, USA). The peritoneum was closed with 3/0 polyglycolic acid sutures (Vicryl^®^, Ethicon, USA), and the ports were removed under direct visualization.

A schematic illustration of port placement and working angles may further facilitate the understanding of the technique; however, such visual materials were not available in the present retrospective study.

### Statistical analysis

In this study, unilateral and bilateral inguinal hernia patients were evaluated separately to determine the optimal cut-off values for the umbilicus–symphysis pubis (USP) distance. The Maximally Selected Rank Statistics (MaxStat) method was used to determine the cut-off points, and the aim was to identify the threshold values that provided the most significant distinction in terms of operating time for the USP distance. Analyses were performed using the MaxStat library in the Python 3.11.7 programming language.

Based on the cut-off values obtained from the MaxStat analysis, patients were divided into two groups: those with USP distances equal to or below the cut-off value and those above the cut-off value. The operating times between these two groups were compared. Due to the non-normal distribution of the data, intergroup comparisons were performed using the non-parametric Mann–Whitney U test with IBM SPSS Statistics version 26.0 (IBM Corp., Armonk, NY, USA). A *p* < 0.05 value was considered statistically significant.

## Results

A total of 341 patients who met the inclusion and exclusion criteria were included in the study. Of these patients, 272 underwent surgery for unilateral inguinal hernia and 69 for bilateral inguinal hernia. The age range of the study population was between 18.00 and 77.00 years, with a median age of 44.00 years. The baseline demographic and clinical characteristics of the study population are summarized in Table 1. The majority of patients had unilateral hernias (79.8%), while 20.2% had bilateral hernias. Among unilateral cases, right-sided hernia was more common (203 vs. 69). The median BMI was 28.5 kg/m². The median USP distance was 17.30 cm (range: 9.70–23.50 cm).

When evaluating the umbilicus–symphysis pubis (USP) distance, a significant anatomical variation was observed among patients. In unilateral cases, the USP distance ranged from 9.70 cm to 23.50 cm, while in bilateral cases, this value ranged from 11.10 cm to 22.30 cm. The relationship between the USP distance and the operation duration was analyzed using the Python-based Maximally Selected Rank Statistics (MaxStat) method, testing different cut-off points. When examining the p-value distribution corresponding to candidate cut-off values, it was observed that the most powerful statistical thresholds for distinguishing operation duration formed distinct peaks. As a result of this analysis, the optimal cut-off value was determined to be 15 cm for unilateral inguinal hernia cases and 16 cm for bilateral cases.

**Table 1:** Comparison of operative outcomes according to USP-based grouping in unilateral and bilateral TAPP repairs

Total patients (*n*)	341
Median age (years)	44.00
Minimum age (years)	18
Maximum age (years)	77
Hernia type	Unilateral: 272 (n) | Bilateral: 69 (n)
Hernia side (unilateral), n (%)	Right: 203 / Left: 69
BMI, median (range), kg/m²	28,5 kg/m²
Median USP distance (cm)	17.30
USP distance range (cm)	9.70–23.50

USP: Umbilicus–Symphysis Pubis distance, n:number, cm: centimeter

**Fig. 2:**
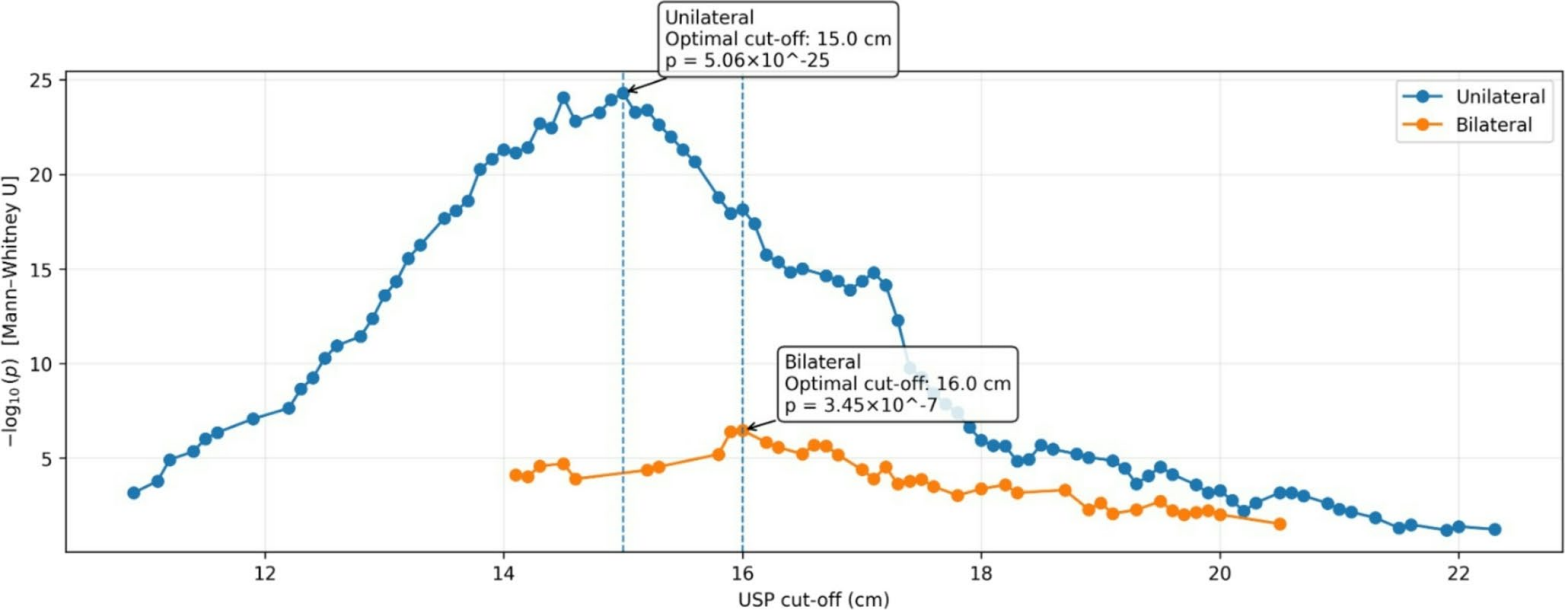
Identification of optimal USP cut-off values using maximally selected rank statistics (MaxStat). The figure shows the distribution of −log10(p) values across tested USP cut-off points obtained using the MaxStat method. Peaks indicate the most discriminative thresholds for operative time. The optimal cut-off values were identified as 15 cm for unilateral and 16 cm for bilateral inguinal hernia cases

These threshold values were then used to form patient groups, and a graph showing the change in the p-value distribution according to the cut-off points is presented in Fig. 2.

Patients were divided into four groups based on the side of the hernia and the USP cut-off values:

- Group 1: Unilateral hernia, USP ≤ 15 cm
- Group 2: Unilateral hernia, USP > 15 cm
- Group 3: Bilateral hernia, USP ≤ 16 cm
- Group 4: Bilateral hernia, USP > 16 cm

The operative times and mean USP values for each group are summarized in Table 2. In unilateral cases, the median operative time was 45.0 min (range: 30.0–65.0) in patients with USP ≤ 15 cm and 32.0 min (range: 18.0–45.0) in patients with USP > 15 cm (*p* < 0.001).

**Table 2:** Demographic and Clinical Characteristics of the Study Population

Unilateral (cut-off = 15.0)	Group 1 (*n* = 60)	Group 2 (*n* = 212)	*p*
Operation duration, median (range), min	45.0 (30.0–65.0)	32.0 (18.0–45.0)	**< 0.001**
Median USP distance, median (range), (cm)	13.40 (9.70–14.90)	17.70 (15.00-23.50)	
Bilateral (cut-off = 16.0)	Group 3 (*n* = 19)	Group 4 (*n* = 50)	p
Operation duration, median (range), min	85.0 (60.0-125.0)	65.0 (30.0–85.0)	**< 0.001**
Median USP distance, median (range), (cm)	14,50 (11.10–15.90)	17.80 (16.00-22.30)	

USP: Umbilicus–Symphysis Pubis distance, n:number, cm: centimeter

When comparing operative times between groups using the Mann–Whitney U test:

- In unilateral cases, a statistically significant difference in operation duration was found between patients with an USP distance of 15 cm or less and those with an USP distance greater than 15 cm (*p* < 0.001).
- Similarly, in bilateral cases, a statistically significant difference in operation duration was found between patients with USP distances ≤ 16 cm and those with USP distances > 16 cm (*p* < 0.001).
- In bilateral cases, the median operative time was 85.0 min (range: 60.0–125.0) in patients with USP ≤ 16 cm and 65.0 min (range: 30.0–85.0) in patients with USP > 16 cm (*p* < 0.001).
- In the unilateral hernia group, it was observed that the operative time was longer in patients with an USP distance ≤ 15 cm and shorter in patients with an USP distance > 15 cm. It was determined that this anatomical effect had the same effect on the operative time in bilateral hernia repairs (≤ 16 cm - >16 cm).

These findings indicate that a shorter USP distance is associated with prolonged operative time in both unilateral and bilateral TAPP repairs.

## Discussion

Laparoscopic surgery has been increasingly applied in many surgical disciplines in recent years and has become one of the fundamental components of modern surgical practice due to the advantages offered by the minimally invasive approach. Gains such as smaller incisions, less postoperative pain, shorter hospital stays, faster return to daily life, and better cosmetic results have led to the widespread adoption of laparoscopic techniques [10, 11]. Parallel to this development, the learning process -required to safely and effectively perform laparoscopic procedures in different surgical fields- has also become an important research topic [12–14].

Surgical difficulty in laparoscopic procedures is influenced by multiple factors, including anatomical variations and the operative field. Previous studies have shown that anatomical proximity of pathological structures to critical surgical landmarks may increase technical complexity, operative time, and the likelihood of conversion to open surgery. In a recent study, it was demonstrated that the location of perforation relative to key anatomical regions was associated with increased surgical difficulty and conversion rates in minimally invasive surgery. These findings support the concept that anatomical parameters may serve as predictive indicators of technical challenge during laparoscopic procedures [15].

Numerous studies have shown that as the surgeon’s experience increases, the duration of surgery decreases, complication rates decrease, and surgical outcomes become more predictable [16, 17]. Today, appropriate patient selection is recognized as an important factor in optimizing surgical outcomes and minimizing operative risk in laparoscopic procedures [18, 19].

Appropriate patient selection is even more critical in laparoscopic pelvic surgeries, particularly where the anatomical working space is limited. The deep and narrow anatomical structure of the pelvic region can directly affect the maneuverability of laparoscopic instruments, the field of view, and the safety of dissection. Therefore, the patient’s anatomical characteristics are among the important factors determining the technical difficulty of the operation. Parameters such as pelvic depth, abdominal wall length, and accessibility to the surgical target area can be decisive in the duration of the operation by affecting the surgeon’s ergonomics and maneuvering capacity. These anatomical variables may contribute to increased technical difficulty and prolonged operative times during laparoscopic pelvic procedures [17, 20, 21].

Inguinal hernia repair is one of the most frequently performed elective surgical procedures by general surgeons [1, 2]. Given this frequency, preoperatively anticipating anatomical factors that may influence operative complexity could contribute to more informed surgical planning and resource allocation.

The exclusion criteria applied in the study aimed to create a more homogeneous patient group by eliminating, as far as possible, other variables that could affect the operative time and technical difficulty.

Female patients were excluded from the study to reduce anatomical heterogeneity and to obtain a more homogeneous study population for evaluating the relationship between USP distance and operative time. This exclusion was not intended to imply that female anatomy is less complex, but rather to minimize the potential confounding effect of sex-related anatomical differences in the inguinal region.

Recurrent inguinal hernia cases are known to be technically more challenging due to fibrotic changes in the dissection area associated with previous surgical interventions, disruption of tissue planes, and adhesions, particularly to previously placed mesh material. As surgical dissection in these patients is a more careful and experience-requiring process, which could significantly affect the duration of the operation, recurrent cases were not included in the study.

In patients with a high body mass index (BMI > 35 kg/m²), achieving adequate exposure may be difficult due to thickened abdominal wall and increased intra-abdominal fat tissue, making it challenging to clearly visualize anatomical structures and potentially prolonging the operative time. Therefore, obese patients were also excluded to obtain a homogeneous study group.

In patients with scrotal hernias, dissection of the hernial sac and safe reduction of its contents into the abdomen is a technically more difficult and experience-requiring process. Large hernial sacs, long-standing hernias, and the presence of scrotal extension may increase the duration and difficulty of dissection; therefore, these cases were excluded from the study.

The risk of intraoperative and postoperative bleeding is significantly increased in patients receiving anticoagulant therapy, requiring more controlled and careful dissection. As the additional interventions required for bleeding control may affect the duration of the operation, this patient group was also excluded from the study.

Patients requiring emergency surgery present a different surgical dynamic compared to elective cases due to additional difficulties arising from their clinical condition, oedematous tissues, strangulation findings, and limited preoperative preparation time. As this situation may directly affect the duration of the operation and the degree of technical difficulty, emergency cases were excluded from the study.

Cases converted to open surgery due to intraoperative complications were excluded from the evaluation because the planned laparoscopic procedure could not be completed. In these cases, the operative time and technical difficulty did not directly reflect the laparoscopic dissection process; therefore, they were not included in the analyses to preserve the methodological integrity of the study.

The findings obtained in this study reveal noteworthy results when compared to studies examining the effect of anatomical factors on operation duration and technical difficulty in laparoscopic surgeries, thereby contributing to the literature. One study in the literature evaluated unilateral TAPP repair in 80 patients and bilateral TAPP repair in 42 patients; it reported that the USP distance did not significantly affect the operative time in unilateral hernia repairs, whereas in bilateral repairs, the operative time increased significantly as the USP distance increased [5]. In contrast to these findings, our study demonstrated that operative times were significantly longer in patients with shorter USP distances in both unilateral (≤ 15 cm) and bilateral (≤ 16 cm) cases.

This suggests that the USP distance may be an anatomical marker that can affect the operative time not only in bilateral cases but also in unilateral surgeries when appropriate threshold values are considered. This difference may be attributed to variability in the patient population, sample size, and surgical application dynamics.

From an ergonomic perspective, laparoscopic TAPP repair is highly dependent on appropriate triangulation and sufficient working distance between ports. A shorter umbilicus–symphysis pubis distance may reduce the effective working space in the lower abdomen, leading to increased instrument collision (swording), restricted movement angles, and technical challenges during dissection and peritoneal closure. These limitations may be particularly pronounced when the camera port is placed infraumbilically, as this configuration reduces the distance between the optical axis and working instruments, thereby negatively affecting visualization and maneuverability.

It should be noted that the ergonomic impact of camera port positioning may vary depending on surgical preference and technique, and infraumbilical placement, although standard in our practice, may present technical challenges such as instrument crowding in certain cases. Additionally, alternative anatomical measurements, such as the distance between the xiphoid process and the umbilicus, may also be considered in surgical planning; however, these were not evaluated in the present study and may be explored in future research.

The importance of anterior abdominal wall anatomy in planning appropriate port placement for laparoscopic surgery has been emphasized in numerous studies [20, 21]. The distance between the umbilicus and the pelvis, abdominal wall length, and anatomical features of the lower abdomen are among the parameters that can directly affect the surgeon’s working angle and instrument movement freedom. It is noted that, particularly in procedures targeting the pelvic region, planning port placement based on patient-specific anatomical features may contribute to a more effective and controlled execution of the operation.

However, a study evaluating the TEP technique reported that anatomical measurements of the anterior abdominal wall did not show a significant correlation with operating time [6]. This finding indicates that anatomical parameters may not have the same impact across different surgical techniques. In the present study, the significant association observed between USP distance and operative time in both unilateral and bilateral cases using the TAPP approach suggests that this difference may be related to the characteristics of the surgical technique itself. While the TEP procedure involves limited dissection within a confined extraperitoneal space, the TAPP technique requires intraperitoneal access and a wider dissection field. Consequently, anatomical parameters reflecting anterior abdominal wall length may have a more pronounced influence on working angles, instrument maneuverability, and overall operative performance in TAPP repair.

The umbilicus–pubic symphysis distance represents a simple and easily measurable anatomical parameter that can be obtained preoperatively without the need for additional equipment, making it clinically practical. Patients with a shorter USP distance were associated with longer operative times in the present study, which may be explained by reduced working space and increased instrument interference. However, this parameter should not be interpreted as a standalone determinant of technical difficulty, but rather as a supportive anatomical indicator within a multifactorial framework. Operative time is influenced by multiple factors, including hernia type, sac size, hernia side, abdominal wall morphology, and disease duration, and therefore cannot be attributed to a single anatomical parameter alone. Accordingly, USP distance may be considered as a complementary factor in preoperative assessment and surgical planning, rather than a definitive predictor of operative difficulty. In practical terms, USP distance measurement requires no additional equipment or imaging and can be performed within seconds during routine physical examination. Surgeons may use this information to anticipate potentially longer operative times, optimize operating room scheduling, and prepare for cases that may require additional technical attention. These applications do not alter the indication for surgery but may support more informed and efficient perioperative planning.

In addition, no radiological or imaging-based anatomical measurements were included in this study. Although such evaluations could provide a more comprehensive assessment of pelvic anatomy and surgical difficulty, the aim of this study was to focus on a simple, clinically applicable parameter that can be obtained during routine physical examination.

## Conclusion

This study evaluated the relationship between the umbilicus–symphysis pubis (USP) distance and the duration of laparoscopic inguinal hernia repair performed via TAPP and demonstrated that this anatomical measurement could be a parameter associated with operative duration and procedural complexity in both unilateral and bilateral cases. These findings suggest a potential association between USP distance and operative complexity; however, external validation in prospective and multicenter studies is needed before this parameter can be recommended for routine preoperative assessment.

The study has some limitations. Firstly, its single-center and retrospective design may limit the generalizability of the results. Furthermore, other limitations include inter-surgeon technical differences that may affect operating times, other variables related to patient anatomy, the absence of additional ergonomic and radiological parameters, the lack of pelvic anatomical measurements that could have provided a more comprehensive evaluation of surgical ergonomics, and the absence of an objective scoring system that directly measures the degree of dissection difficulty. Additionally, the analysis was based on a single anatomical parameter without adjustment for potential confounders such as hernia type, sac size, disease duration, patient morphology, and surgeon-related variables. The absence of a multivariable analysis represents an important methodological limitation, and future studies should incorporate such analyses to better isolate the independent contribution of USP distance to operative time. The lack of radiological or imaging-based anatomical assessment may further limit the comprehensive evaluation of pelvic anatomy and its impact on surgical difficulty. Importantly, the highly selected nature of the study population, resulting from extensive exclusion criteria, may limit the generalizability of these findings to the broader and more heterogeneous TAPP patient population encountered in routine clinical practice. However, the fact that the USP distance was measured and recorded prospectively and that a homogeneous patient group was evaluated can be considered methodological strengths of the study.

Appropriate patient selection is a key component of operative planning in laparoscopic surgery. The findings of the present study suggest that USP distance may serve as a simple, preoperatively measurable parameter that could support case planning by helping surgeons anticipate longer operative times. However, this hypothesis requires prospective validation before USP distance can be recommended as a routine patient selection criterion.

## Data Availability

The database of this study is open to sharing. It can be obtained from the authors upon request.

## Declarations

### Scientific responsibility statement

The authors declare that they are responsible for the scientific content of the article, including study design, data collection, analysis and interpretation, writing, part or all of the outline, preparation and scientific review of the content, and approval of the final version of the article.

## References

[1] HerniaSurge Group (2018) International guidelines for groin hernia management. Hernia 22(1):1–165 10.1007/s10029-017-1668-x PMC5809582 29330835

[2] Stabilini C, van Veenendaal N, Aasvang E, Agresta F, Aufenacker T, Berrevoet F, Burgmans I, Chen D, de Beaux A, East B, Garcia-Alamino J, Henriksen N, Köckerling F, Kukleta J, Loos M, Lopez-Cano M, Lorenz R, Miserez M, Montgomery A, Morales-Conde S, Oppong C, Pawlak M, Podda M, Reinpold W, Sanders D, Sartori A, Tran HM, Verdaguer M, Wiessner R, Yeboah M, Zwaans W, Simons M (2023) Update of the international HerniaSurge guidelines for groin hernia management. BJS Open 7(5):zrad08037862616 10.1093/bjsopen/zrad080PMC10588975

[3] Miserez M, Peeters E, Aufenacker T, Bouillot JL, Campanelli G, Conze J, Fortelny R, Heikkinen T, Jorgensen LN, Kukleta J, Morales-Conde S, Nordin P, Schumpelick V, Smedberg S, Smietanski M, Weber G, Simons MP (2014) Update with level 1 studies of the European Hernia Society guidelines on the treatment of inguinal hernia in adult patients. Hernia 18(2):151–16324647885 10.1007/s10029-014-1236-6

[4] Daes J, Felix E (2017) Critical view of the myopectineal orifice. Annals ofSurgery 266(1):e1–e2 10.1097/SLA.0000000000002104 27984213

[5] Arıcı S (2023) Impact of umbilicus–symphysis pubis distance on technical difficulties in transabdominal preperitoneal hernia repair (TAPP). Surg Laparosc Endosc Percutan Tech 33(5):511–51437725830 10.1097/SLE.0000000000001220

[6] Meyer A, Blanc P, Kassir R, Atger J (2014) Laparoscopic hernia: Umbilical–pubis length versus technical difficulty. JSLS: J Soc Laparoendoscopic Surg 18(3):e201400078 10.4293/JSLS.2014.00078 PMC4208897 25392661

[7] Bracale U, Merola G, Sciuto A, Cavallaro G, Andreuccetti J, Pignata G (2019) Achieving the learning curve in laparoscopic inguinal hernia repair by TAPP: A quality improvement study. J Invest Surg 32(8):738–74529902096 10.1080/08941939.2018.1468944

[8] Khan N, Abboudi H, Khan MS, Dasgupta P, Ahmed K (2014) Measuring the surgical learning curve. BJU Int 113(3):504–50823819461 10.1111/bju.12197

[9] Bökeler U, Schwarz J, Bittner R et al (2013) Teaching and training in laparoscopic inguinal hernia repair (TAPP): Impact of the learning curve on patient outcome. Surg Endosc 27:2886–289323436092 10.1007/s00464-013-2849-z

[10] Brian R, Davis G, Park KM, Alseidi A (2022) Evolution of laparoscopic education and the laparoscopic learning curve: A review of the literature. Laparosc Surg 6:34

[11] Maheshwari S, Sharma BK, Misra MC (2021) Learning curve in laparoscopic inguinal hernia repair. J Mahatma Gandhi Univ Med Sci Technol 6(2):48–52

[12] Ichikawa N, Homma S, Yoshida T, Emoto S, Imaizumi K, Matsui H, Tani M, Miyaoka Y, Taketomi A (2022) Interischial spine distance is a simple index of the narrow pelvis that can predict difficulty during laparoscopic low anterior resection. Surg Laparosc Endosc Percutan Tech 32(6):666–67236223301 10.1097/SLE.0000000000001111

[13] Fung ACH, Chan IHY, Wong KKY (2023) Outcome and learning curvefor laparoscopic intracorporeal inguinal hernia repair in children. Surg Endosc 37:434–44235986222 10.1007/s00464-022-09530-1

[14] Nezhat CH, Nezhat F, Brill AI, Nezhat C (1999) Normal variations of abdominal and pelvic anatomy evaluated at laparoscopy. Obstet Gynecol 94(2):238–24210432135 10.1016/s0029-7844(99)00317-8

[15] Aydoğdu YF, Gülçek E, Koyuncuoğlu AC, Büyükkasap Ç, Dikmen K (2024) Minimally invasive approach in a rare emergency surgery, gallbladder perforation. BMC Surg 24(1):207. 10.1186/s12893-024-02495-z PMID: 38987756; PMCID: PMC1123462138987756 10.1186/s12893-024-02495-zPMC11234621

[16] Muavha DA, Ras L, Jeffery S (2019) Laparoscopic surgical anatomy for pelvic floor surgery. Best Pract Res Clin Obstet Gynaecol 54:89–102 10.1016/j.bpobgyn.2018.11.005 30554856

[17] Kim JY, Kim YW, Kim NK, Hur H, Lee K, Min BS, Cho HJ (2011) Pelvic anatomy as a factor in laparoscopic rectal surgery: A prospective study. Surg Laparosc Endosc Percutan Tech 21(5):334–33922002269 10.1097/SLE.0b013e31822b0dcb

[18] Sivakumar J, Chen Q, Hii MW et al (2023) Learning curve of laparoscopic inguinal hernia repair: Systematic review, meta-analysis, and meta-regression. Surg Endosc 37:2453–247536416945 10.1007/s00464-022-09760-3

[19] Köckerling F, Bittner R, Kraft B et al (2017) Does surgeon volume matter in the outcome of endoscopic inguinal hernia repair? Surg Endosc 31:573–58527334968 10.1007/s00464-016-5001-zPMC5266765

[20] Akesson C, Mintz G, Sridhar S, Gubbels AC (2025) Precise port placement: An analytic review of lower quadrant anatomy. J Minim Invasive Gynecol 32(11 Suppl):S21

[21] Proietti F, La Regina D, Pini R, Di Giuseppe M, Cianfarani A, Mongelli F (2021) Learning curve of robotic-assisted transabdominal preperitoneal repair (rTAPP) for inguinal hernias. Surg Endosc 35(12):6643–664933258030 10.1007/s00464-020-08165-4

